# A Tumor initiating cell-enriched prognostic signature for HER2^+^:ERα^−^ breast cancer; rationale, new features, controversies and future directions

**DOI:** 10.18632/oncotarget.1170

**Published:** 2013-07-26

**Authors:** Jeff C. Liu, Sean E. Egan, Eldad Zacksenhaus

**Affiliations:** ^1^ Division of Cell & Molecular Biology, Toronto General Research Institute - University Health Network, Toronto, Canada; ^2^ Program in Developmental and Stem Cell Biology, The Hospital for Sick Children, Toronto

**Keywords:** HER2 breast cancer, Prognostic signature, Tumor initiating cell, Cancer stem cells, Mouse models

## Abstract

The high intra- and inter-tumor heterogeneity of many types of cancers, including breast cancer (BC), poses great challenge to development of subtype-specific prognosis. In BC, the classification of tumors as either ERα^+^ (Luminal A and Luminal B), HER2^+^ (ERα^+^ or ERα^−^) or triple-negative (TNBC)(Basal-like, claudin-low) guides both prognostication and therapy. Indeed, prognostic signatures for ERα^+^ BC are being incorporated into clinical use. However, these signatures distinguish between luminal A (low risk) and Luminal B (high risk) BC; signatures that identify low/high risk patients with luminal B BC are yet to be developed. Likewise, no signature is in clinical use for HER2^+^ or TNBC. The major obstacles to development of robust signatures stem from diversity of BC, clonal evolution and heterogeneity within each subtype. We have recently generated a prognostic signature for HER2^+^:ERα^−^ BC based on the identification of genes that were differentially expressed in a tumor-initiating cell (TIC)-enriched fraction versus non-TIC fraction from a mouse model of HER2^+^ BC (MMTV-Hers/Neu). Here we describe the rationale behind development of this prognosticator, and present new features of the signature, including elevated PI3K pathway activity and low TNFalpha and IFNgamma signaling in high-risk tumors. In addition, we address controversies in the field such as whether random gene expression signatures significantly associate with cancer outcome. Finally, we suggest a guideline for development of prognostic signatures and discuss future directions.

## INTRODUCTION

Breast cancer (BC) is a heterogeneous disease that includes ERα^+^ (~65%; luminal A and B), HER2^+^ (~10% HER2^+^:ERα^+^; ~10% HER2^+^:ERα^−^), and triple negative (~15%; Basal-like, Claudin-low, metaplastic) forms [1]. Luminal A tumors have the best prognosis followed by luminal B and HER2^+^:ERα^+^, with HER2^+^:ERα^−^, Basal-like and Claudin-low having the worst outcome. Patients with ERα^+^ tumors are treated with tamoxifen and aromatase inhibitors [2], HER2^+^ tumors with chemotherapy plus anti-HER2 antagonists such as trastuzumab, a monoclonal antibody directed against HER2 [3-5], whereas TNBCs are currently treated with chemotherapy alone [6]. Many of these tumors may not develop macro-metastases, and therefore surgical removal alone with local radiation or hormonal therapy can cure patients. In contrast, other tumors form distal metastases that are virtually incurable. The goal of prognostic signatures is to segregate patients with primary tumors into low and high-risk groups, thereby identify patients who would benefit from withholding harsh therapy and those who will benefit from aggressive intervention.

Heterogeneity within a tumor can affect its dissemination potential and response to therapy. In addition, many types of cancer exhibit hierarchical organization whereby only a fraction of cells, termed cancer stem cells (CSC) or, as they are operationally defined by transplantation assays, Tumor Initiating Cells (TICs), sustains growth, whereas the remaining tumor cells, which descend from TICs, have lost their tumorigenic potential [7]. Early prognostic signatures were developed irrespective of this hierarchy or the diversity of BC. These signatures, such as Oncotype [8], predict outcome for ERα^+^ BC, the majority of cases, but not for HER2^+^ or triple negative BC [9]. These signatures also seem to segregate Luminal A (low proliferation, low risk) from Luminal B (high proliferation, high risk) patients, but not good/bad prognosis within each subtype. A second type of prognostic signature was developed for cancer stem cells (CSC/TICs). Thus, an invasiveness gene signature (IGS) was generated from CD44^+^/CD24^−/low^ breast TICs isolated from Pleural effusions from patients with metastatic disease [10]. When analyzed against several independent cohorts, this signature scored modestly for ERα^+^ BC (HR, 2.12) but poorly for TNBC (HR, 1.08) and HER2^+^ (HR, 0.96) patients [8]. A third type of prognostic signature was designed for specific breast cancer subtypes but irrespective of tumor hierarchy. This includes a stromal-derived prognostic predictor (SDPP) [11], which we found to be more predictive for HER2^+^:ERα^+^ than for HER2^+^:ERα^−^ BC [12]. A signature has recently been developed for TNBC, based on microarray data from whole tumors [13]. A molecular signature of normal breast epithelial and stromal cells from Li-Fraumeni syndrome with p53 mutations has also been established [14]. Whether it predicts clinical outcome for TNBC or HER2^+^ BC patients, in which p53 is frequently inactivated, remains to be seen. For a more detailed discussion of prognostic signatures, mainly for ERα^+^ BC, we refer readers to excellent recent reviews [15, 16]. Importantly, none of these signatures were developed on the basis of TICs for a specific BC subtype. Here we discuss development of a TIC-derived signature for HER2^+^:ERα^−^ BC, some new features of the signature, and lessons learned in this process.

### Generation of a TIC-enriched prognostic signature for HER2^+^:ERα^−^ BC

Cognizant of the complexity of human BC, we have sought to derive a prognostic signature for HER2^+^:ERα^−^ BC using TICs from this subtype. As we can use transplantation into immune-competent host to operationally define TICs in the mouse, we chose to analyze tumors from MMTV-Her2/Neu mice, which give rise to HER2^+^:ERα^−^-like mammary tumors, for these studies. We first identified through transplantation assays HER2^+^:ERα^−^ TICs as CD24^+^:JAG^−^ [12]. TIC frequency was ~2-4.5%. Next, using differentially expressed genes between the TIC-enriched fraction and non-TIC fraction we developed a 17-gene Her2-TIC-enriched signature (HTICS) that predicted clinical outcome on several independent HER2^+^ cohorts. Its prognostic power was independent of other predictors, stratified lymph node^+^ HER2^+^ BC into low- and high-risk subgroups, and was specific for HER2^+^:ERα^−^ patients (hazard ratio (HR)=5.57; P=0.002)). Retrospective analyses revealed that patients with HTICS^+^ HER2^+^:ERα^−^ tumors resisted chemotherapy but responded to chemotherapy plus trastuzumab [12]. HTICS is therefore a promising new prognostic signature for HER2^+^:ERα^−^ BC that may be used to identify high-risk patients that would benefit from anti-HER2 therapy.

Notably, a substantial percentage of HTICS^+^:HER2^+^:ERα^−^ patients still developed metastasis (27%) or died (22%) within 4 years post-surgery even when treated with trastuzumab. HTICS^+^ patients may relapse because they are inherently resistant to trastuzumab and/or are prone to become drug-resistant. HER2^+^ TICs depend on HER2 signaling [17]. Therefore HTICS^+^ tumors should exhibit increased sensitivity to trastuzumab. On the other hand, in HTICS^+^ patients, a high frequency of TICs with enhanced self-renewal capacity may facilitate the accumulation of new mutations or epigenetic changes that can lead to clones with increased drug resistance (e.g. SRC activation [18]. Alternatively, HTICS^+^ tumors may represent a distinct, more aggressive subtype of HER2^+^:ERα^−^ BC (see below).

### HTICS: proliferation and immune response genes

**Figure 1:**
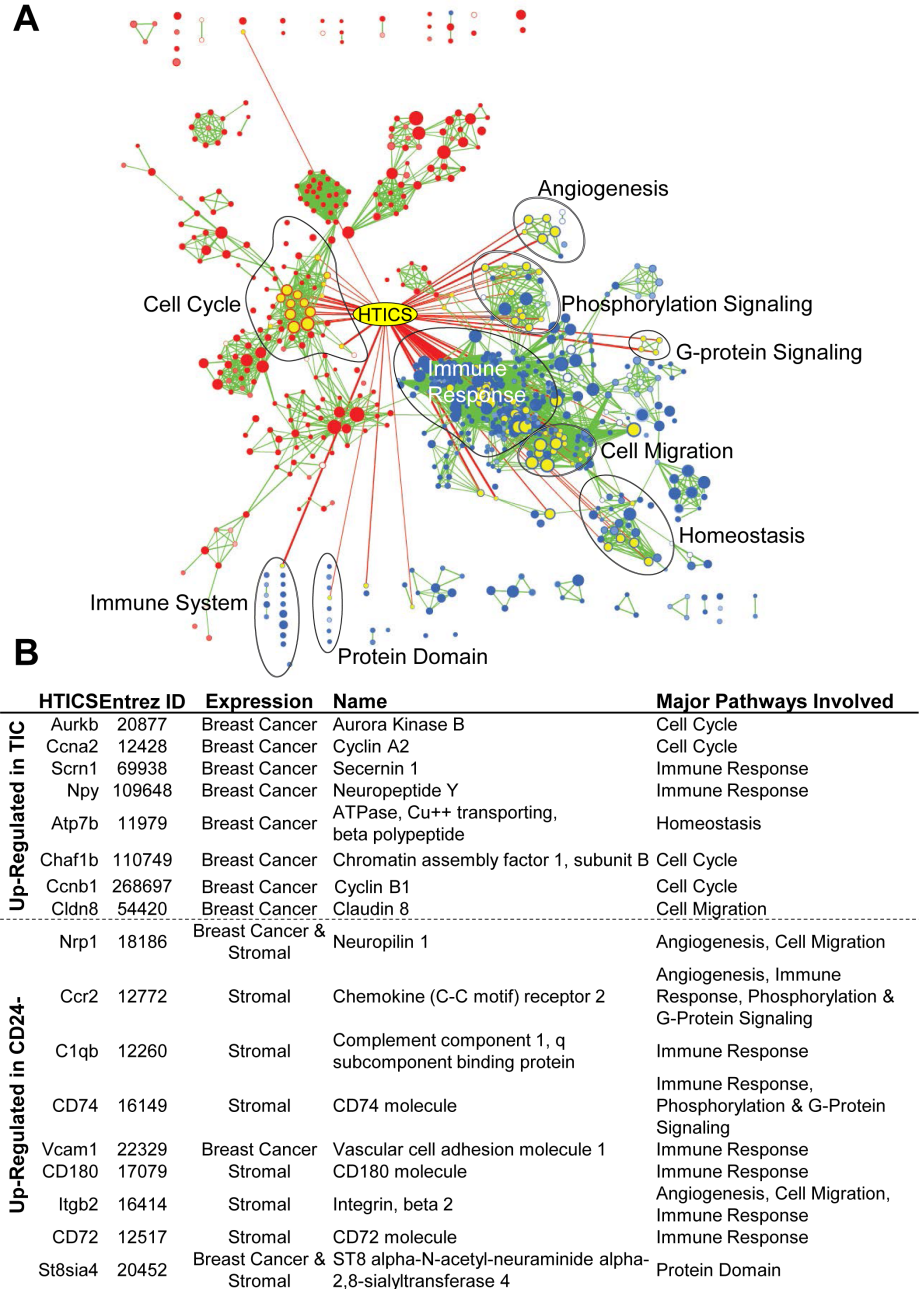
Pathway analysis of HTICS (A) The HTICS genes are listed with Entrez ID, Expression in different cell types as determined by analysis of HER2^+^ JIMT1, HCC1954 and BT549 breast cancer cells as well as Human Peripheral Blood Mononuclear Cells, official name, and pathways involved (as per panel B). (B) Superimposed HTICS genes onto GSEA pathways resulting from comparison between TIC-enriched and CD24^−^ cell fraction. The node containing HTICS genes is highlighted in yellow. Pathways highly involved with HTICS are circled and labeled.

HTICS included 8 up-regulated (Aurkb, Ccna2, Scrn1, Npy, Atp7b, Chaf1b, Ccnb1, Cldn8) and 9 down-regulated genes (Nrp1, Ccr2, C1qb, Cd74, Vcam1, Cd180, Itgb2, Cd72, St8sia4). Pathway analysis of these genes, shown in Fig. 1A-B, classifies HTICS genes into 4 groups (i) cell cycle progression (Aurkb/Aurora kinase B, Ccna2/CyclinA2, Ccnb1/cyclinB1, Chaf1b/chromatin Assembly Factor 1 Subunit B); (ii) immune-response (Scrn1, Npy, C1qb, CD74, Vcam1, CD180, CD72); (iii) angiogenesis (Nrp1, Ccr2, Itgb2); and (iv) others (Atp7b, Cldn8, St8sia4). Some of these genes, e.g. Aurora kinase B, are potential therapeutic targets.

We have recently performed gene expression analysis on human (HER2+ and Basal) BC cell lines, and confirmed expression of 11 HTICS genes (Aurkb, Ccna2, Scrn1, Npy, Atp7b, Chaf1b, Ccnb1, Nrp1, Vcam1, Itgb2, and St8sia4; Fig. 1B). In contrast, analysis of human peripheral blood monocyte cells revealed expression of Nrp1, Ccr2, C1qb, CD74, CD180, CD72, and St8sia4. The composition of HTICS is therefore consistent with previous reports that improved clinical outcome for HER2^+^ BC patients correlates strongly with immune response [19, 20]. HTICS may be powerful because it encompasses both proliferation and immune-response/stromal genes.

The identification of immune related genes in HTICS is somewhat surprising given that the signature was developed from lineage-depleted TICs. Indeed, the generation of a prognostic signature from TICs has the disadvantage that tumor-associated stroma cells, which express many informative markers, are intentionally depleted in the course of purifying TICs. In accordance, Morag Park and colleagues have generated potent prognostic signatures for breast cancer on the basis of cancer-associated stroma [11]. In MMTV-Her2/Neu tumors, we found that the CD24^+^:JAG1^−^ population was enriched for TICs, whereas the CD24^−^:JAG1^−^ double-negative population contained both mammary epithelial and stroma cells including immune-cells. The latter was evident from gene expression profiling and pathway analysis [12] (Fig. 1). Thus, the presence of contaminating stroma in the double-negative fraction inadvertently contributed to robustness of our signature.

### Ruling out randomness

**Figure 2:**
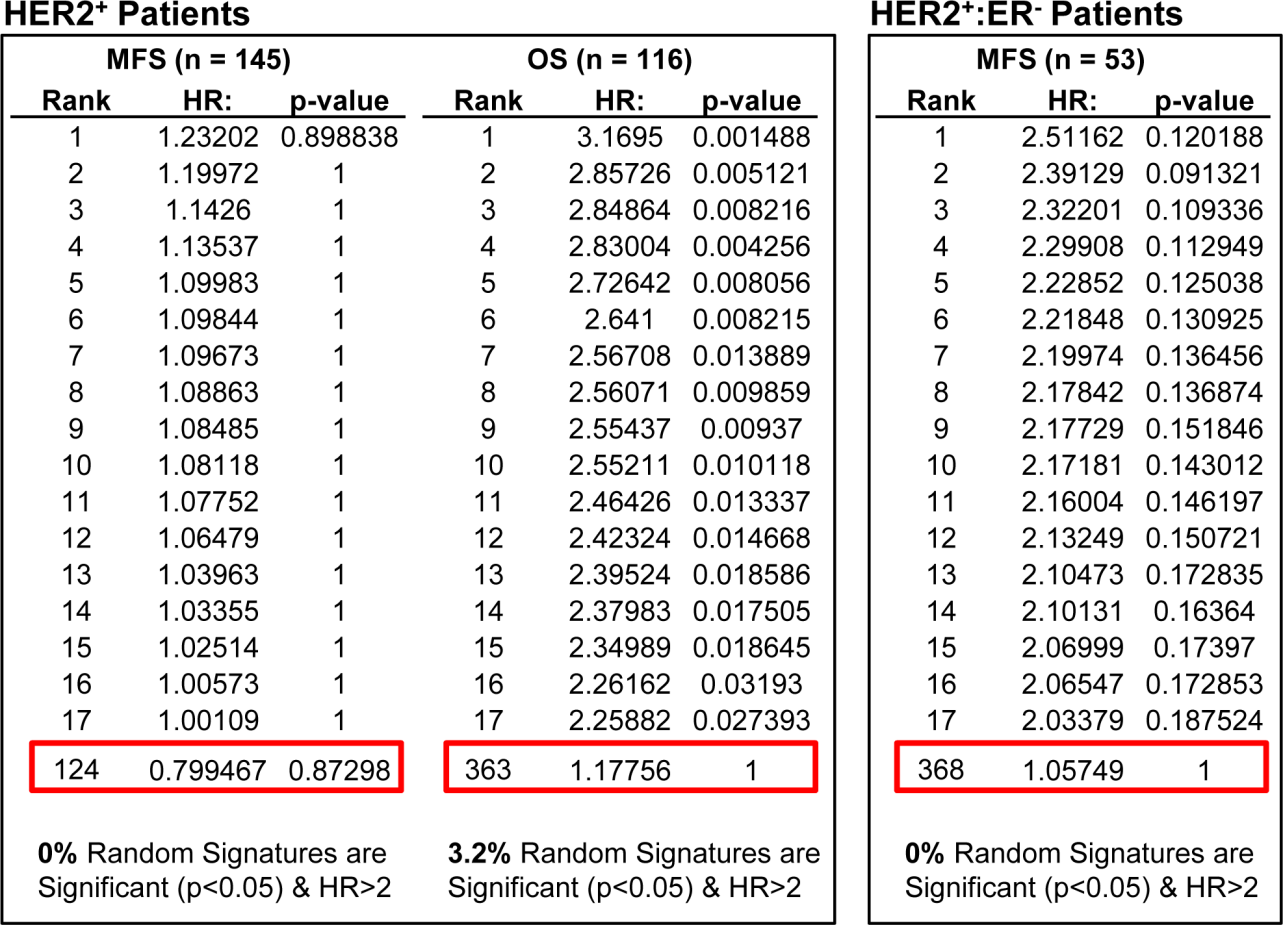
Weak prognostic power of “Mouse Social Defeat Signature” compared with 1000 random signatures in HER2+ breast cancer patients Of the 163 genes in the Mouse Social Defeat (MSD) signature, 148 were found on GPL570 and 127 on GPL96 platforms. Analysis was performed with 116 HER2^+^ patients from 2 GPL570 cohorts (GSEs 16446 and 20685 with Overall Survival (OS) data) and 145 HER2^+^ patients from 6 GPL96 cohorts (GSEs 2034, 2603, 5327, 6532, 11121, and 25066 with Metastasis-Free Survival (MFS) data). The MFS cohorts contain 53 HER2^+^:ER^−^ patients, sufficient for independent analysis. A set of 1000 random signatures with the same number of genes was generated from atmosphere background noise (random.org) for the OS analysis, and another set for MFS. We then used SSM algorithm (assuming all genes are up-regulated) to differentiate samples in OS and MFS cohorts. The signatures were ranked by HR and compared with Mouse Social Defeat Signature (red boxes). The % signatures with significant HR > 2.0 are listed at the bottom. Comparing to 1000 sets of random signatures, Mouse Social Defeat Signature ranked 124 for HER2^+^ MFS samples, 363 for HER2^+^ OS samples, and 368 for HER2^+^:ER^−^ MFS samples.

A recent report by Venet and colleagues state that most random gene expression signatures are significantly associated with breast cancer outcome [21]. Specifically, 3 unrelated signatures (e.g. “social defeat in mice”) were shown to be significantly associated with clinical outcome of breast cancer patients (P < 0.05). However, the HRs predicted by these examples were low (2.4, 1.9 and 1.8). Moreover, the signatures were not directed, i.e. both positive and negative correlations were considered informative – whereas biological signatures are directed (i.e. HTICS^+^ correlates with poor prognosis). This constraint would reduce random correlation by a factor of 2. In addition, for signatures that comprise up-regulated and down-regulated genes, randomness in a specific direction would be reduced by another factor of 2. Finally, the Venet *et al*. manuscript tested for prognostic value of the 3 unrelated signatures against only one cohort (NKI). But for a signature to be valuable, it must be able to predict outcome on multiple independent cohorts. Thus, if a signature has a genuine biological basis, it will predict clinical outcome on independent cohorts whereas a randomly selected signature that performs well on one cohort will fail on others. Indeed, using a computer algorithm to optimize signatures on a training cohort, we identified signatures for HER2^+^ BC with HR of over 20, which were completely uninformative when tested against other cohorts. To directly test our prediction, we analyzed the “social defeat in mice” signature from Venet *et al*. (which gave HR of 2.4 on the NKI cohort), on multiple BC cohorts. This gave HR of approximately ~0.8, 1.2 and 1.1 (Fig. 2), demonstrating that at least this randomly selected signature performs poorly when tested on independent cohorts.

**Figure 3:**
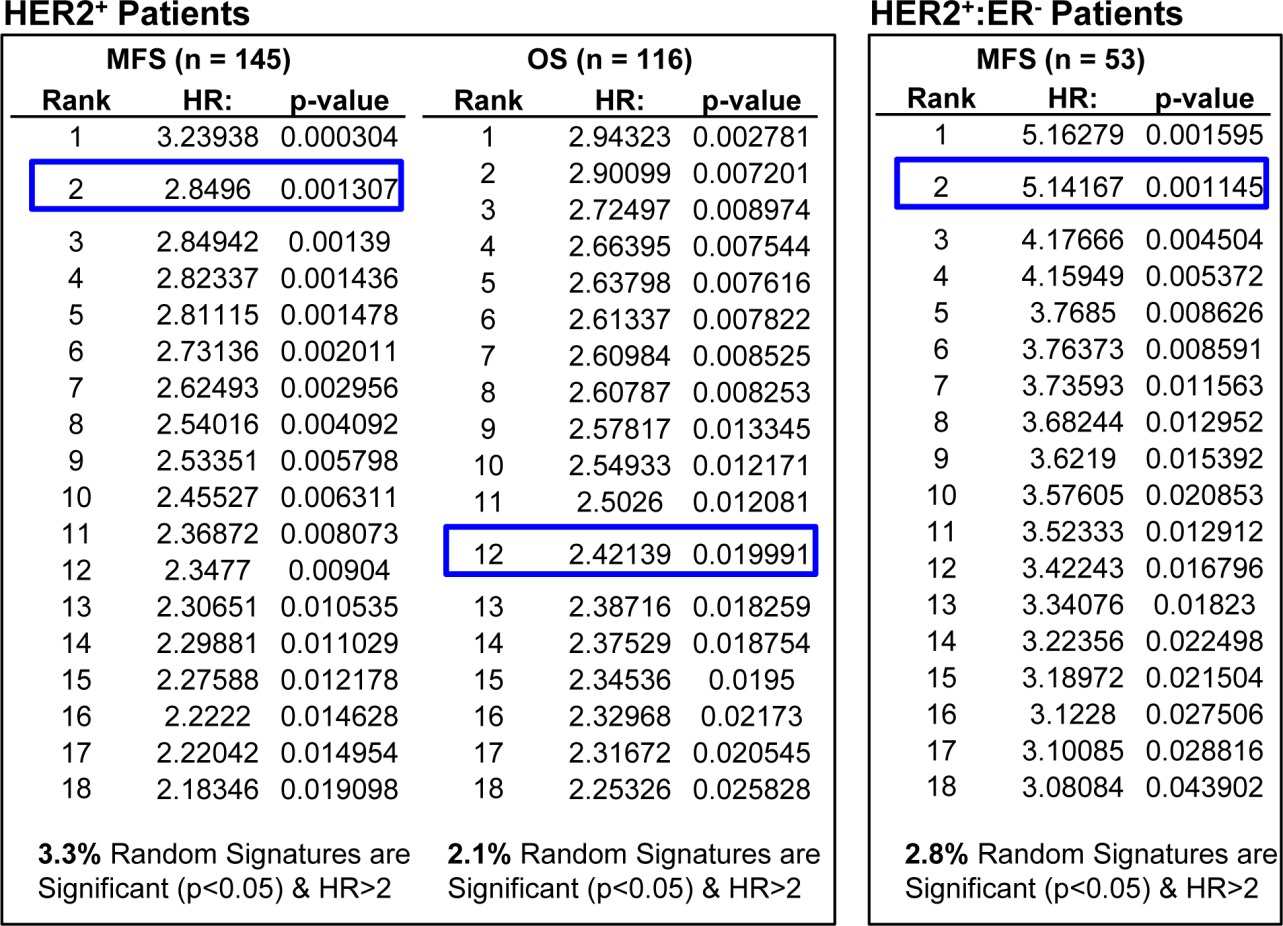
Comparing the prognostic power of HTICS with 1000 random sets of signatures in HER2+ breast cancer patients All HTICS 17 genes (8 up-regulated and 9 down-regulated) were present on both GPL570 and GPL96 platforms. Analysis was performed with 116 HER2^+^ patients from 2 GPL570 and GPL96 cohorts (see legends to Figure 2). A set of 1000 random signatures with the same number of genes (8 genes up-regulated, 9 down-regulated) were generated from atmosphere background noise (random.org) for OS analysis, and another for the MFS, and then SSM algorithm was employed to differentiate samples in OS and MFS cohorts. The signatures were ranked by HR and compared with HTICS (blue boxes). The % of signatures with significant HR > 2.0 is listed at the bottom. Comparing to 1000 sets of random signatures, HTICS ranked 2^nd^ for HER2^+^ MFS, 12^th^ for HER2^+^ OS, and 2^nd^ for HER2^+^:ER^−^ MFS.

To test for significance of a candidate signature, Venet *et al*., suggested to test 1000 random signatures of the same composition against the same cohorts, and determine whether less than 5% of these random signatures (i.e. p< 0.05) have similar HR. For the “social defeat in mice”, comparison to 1000 random sequences of similar structure ranked this signature at 124, 363 and 368 (Fig 2). In contrast, when such analysis was performed on HTICS, we found that only 3.3% of 1000 random signatures (with the same gene composition of HTICS) gave significant HR of more than 2.0, and importantly, HTICS scored highly in all cohorts, e.g. 2^nd^ best on HER2^+^:ERα^−^ patients (Fig. 3).

Thus, for a signature to be valid, both to support a biological process and to predict clinical outcome, we suggest the following guideline:

1. The signature should predict clinical outcome on multiple cohorts with high and significant HR (we suggest >4).
2. The signature should identify a substantial number of patients in high/low risk groups (>10% total) to have clinical utility.
3. A collection of 1000 random signatures of the same size and composition (up- and down-regulated genes) should contain less than 5% of signatures that can predict outcome with similar HR as the candidate signature.

### TIC-derived prognostic signatures and cancer biology

As TICs sustain tumorigenesis, it seems intuitive that a tumor with a high percentage of TICs would be more deadly than a tumor that has relatively fewer TICs. It is important to note however that we used a TIC-enriched fraction, not a pure population of TICs. The percentage of TICs in the CD24^+^:JAG1^−^ fraction was still <5% and in most human cancers it is much lower. Thus, the majority of cells in the “TIC-enriched fraction” are non-TICs. Possibly, the actual percentage of TICs may be higher and underestimated due to death of most TICs following transplantation. This view implies that TIC frequency as determined by xenograft assays reflects the hierarchical organization of cancer cells plus the stochastic survival of TICs after transplantation. In this case, the TIC enriched fraction may contain high percentage of TICs, and the signature may indeed reflect the gene expression profile of TICs. Alternatively, the TIC-enriched fraction may comprise low percentage of TICs, and high percentage of TIC-derived progenitors with similar cell surface markers; the signature in this case may reflect the gene expression profile of progenitor cells, which may revert at low frequency back to TICs [22].

There is strong evidence that the rate-limiting step in metastasis is formation of macro-metastases at distal sites, not tumor dissemination (reviewed in [23]). Since we identified TICs through transplantation assays, this population has proven ability to regenerate tumors. Therefore HTICS^+^ tumors may have many TICs, which are more likely to self renew, form micro-metastases at distal sites, and acquire the necessary genetic or epigenetic alterations required for outgrowth of disseminated lesions. Indeed, 4 of 8 up-regulated genes in HTICS are directly involved in cell cycle progression, DNA replication and mitosis (Fig. 1).

### TIC-derived prognostic signatures and TIC biology

The forgoing discussion pertains to tumors driven by a single type of TIC. Clonal and parallel evolution among TICs may complicate generation of TIC-derived prognostic signatures. First, there is evidence that TICs with metastatic potential represent a subclass of TICs. For example, in human pancreatic cancer, TICs express CD133^+^, but only a subclass of CD133^+^:CXCR4^+^ cells located at the invasive front of tumors have metastatic potential [24]. Thus, ideally signatures should be derived from a TIC sub-fraction that contains metastatic initiating cells. Second, the underling assumption of a linear relationship between primary tumors and their metastases is not always correct. Recent sequencing and genetic analyses of paired tumors and metastases reveal that in certain cases, primary tumors and metastases evolve in parallel with the latter arising from a small fraction of primary tumor cells [25, 26]. It remains to be seen whether in such tumors, a transplantation assay would identify the same small fraction of TICs that metastasize in human, i.e. whether the transplantation assay in mice detects cells that metastasize in patients, or whether even tumor cells that do not metastasize in human can score as TICs following injection into immune-compromised mice. In the latter case, it would seem impossible to generate useful prognostic signatures on the basis of enriched TICs.

Third, highly heterogeneous primary tumors may have more than one type of TIC. In such tumors, TICs may be found in different fractions or may have similar cell surface markers hence sorted in the same fraction. In this case, the TIC-enriched signature would be a mixture of 2 different types of TICs. This may reduce (or increase) efficacy of a signature. One way to address this issue is to inject single tumor cells into recipient mice and compare multiple secondary tumors with their parental primary tumor. We performed such analysis on MMTV-Her2/Neu tumors, and found that independent secondary tumors arising from injection of single cells within the TIC-enriched fraction were indistinguishable from primary tumors [12]. These results indicate that MMTV-Her2/Neu tumors have a single type of TIC, and therefore gene expression profiles from enriched TIC fractions can be used to predict the behavior of this BC subtype. Thus, a prerequisite for generation of a TIC-derived signature is the demonstration that the specific tumor subtype has a single type of TIC. Clearly, this prerequisite is difficult to satisfy by single cell transplantation assays for most human tumors, which show much reduced TIC frequency. Together, we suggest that prognostic signatures based on enriched TIC fractions are unlikely to be predictive for cancers that exhibit high heterogeneity, clonal evolution and multiple/distinct TICs. However, for many cancer types with single type of TIC, this approach should yield potent prognostic signatures.

### Interpretation of signature-positive and signature-negative Kaplan-Meier survival curves

**Figure 4:**
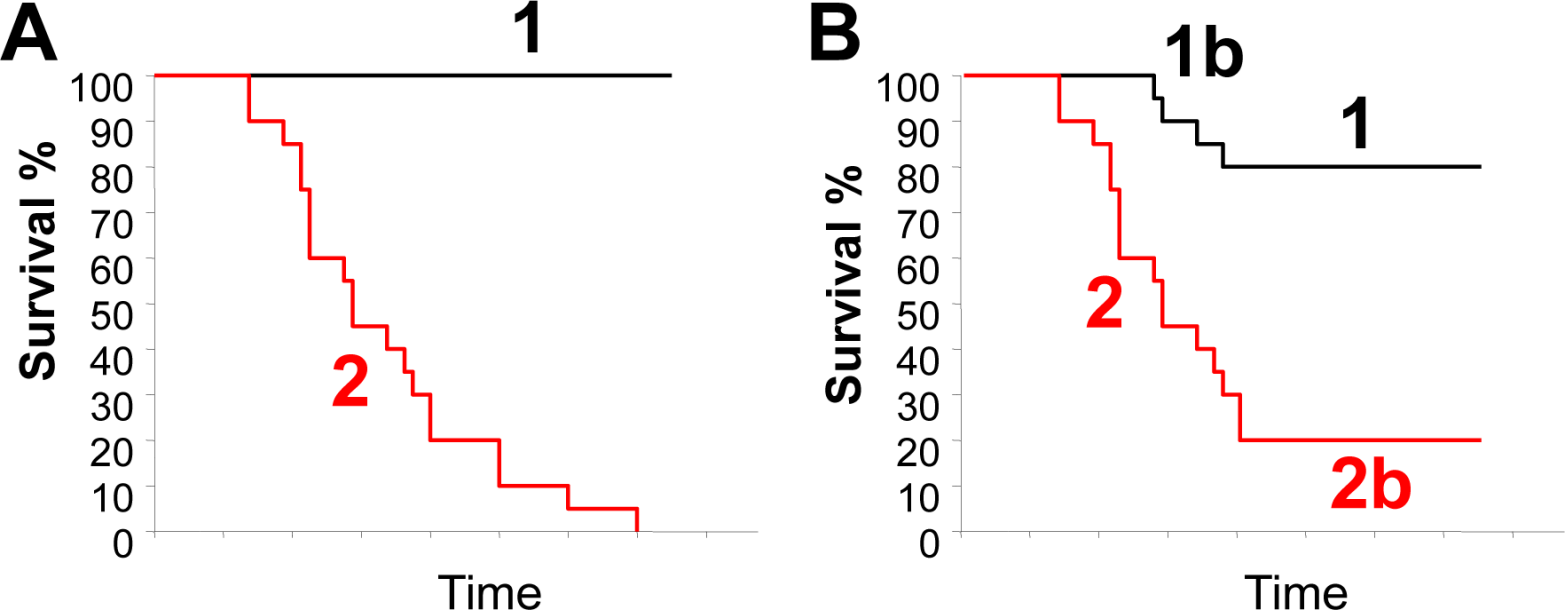
Ideal and typical Kaplan-Meier survival curves of patients segregated on the basis of a prognostic signature (A) An ideal KM curve where all signature-negative patients survive (black line, group 1) and are well separated from signature-positive patients with bad prognosis (red line, group 2). (B) A typical KM curve with signature-negative patients with good prognosis (group 1); signature-positive patients with bad prognosis (group 2); signature-negative but bad prognosis (group 1b); and signature-positive but good prognosis (group 2b). See text for discussion.

Figure 4A depicts idealized and typical Kaplan-Meier (KM) survival curves of patients segregated on the basis of a prognostic signature. In the idealized curve, all signature-negative patients survive (black line), whereas all signature-positive patients die (red line). In such hypothetical curve, signature-negative patients would clearly benefit from receiving minimal or no treatment at all, while signature-positive patients should be treated aggressively. For ERα^+^ BC, many signatures stratify patients with similar KP curves seen in Figure 4A because they identify as signature-negative the luminal-A patients that have excellent prognosis. However, for other BC subtypes, a typical KM survival curve identifies four groups as shown in Figure 4B. Groups 1 and 2 behave as in the idealized curve (Fig. 4A). Yet, group 2 includes patients who succumb to the disease right off the bat and those who die after several years. Tumors from the former group of patients likely have already acquired oncogenic mutations that drive aggressive dissemination/metastasis. In contrast, the latter group may have not yet acquired such mutations at the time of biopsy, but because their tumors have a high number of TICs that self-renew, they are more likely to acquire such mutations that facilitate metastatic disease.

Group 1b represents patients that die despite being signature-negative, whereas group 2b represents patients who survive despite having signature-positive tumors. Survival of group 2b is a welcome outcome; it may reflect successful therapy or timely removal of primary lesions through surgery before tumor cells have disseminated. Group 1b is a major obstacle to clinical use of prognostic signatures because death of signature-negative patients suggests that the entire cohort should be aggressively treated even if most patients in this group would do well with minimal intervention. Thus, to justify withholding aggressive drugs from signature-negative patients, the signature should be predictive enough to render group 1b a small minority. Group 1b may represent a subset of patients that the signature fails to identify as having a poor prognosis. Alternatively, these tumors may be misdiagnosed (e.g. they are ERα^+^ or TNBC not HER2^+^:ERα^−^). It is also possible that these tumors are heterogeneous with some aggressive clones (signature-positive) that were not included or were diluted out in the biopsy from which RNA was extracted for microarray profiling. Alternatively, the tumor may have evolved to become more aggressive (signature-positive) post biopsy. Thus, it is possible that group 1b is unavoidable. The challenge is to generate prognosticators that can separate signature-negative and -positive curves wide enough so that risk of death for group 1b is out-weighted relative to the risk of side effects from aggressive therapy on signature-negative patients. Intriguingly, if HTICS^−^ patients can be classified as a subgroup of HER2^+^:ERα^−^ BC (see next section), there is a possibility that other clinical parameters or signatures could further separate group 1b and help identify novel treatments for these patients.

### Are signature-negative and signature-positive tumors close variants or different subtypes?

Elevated PI3K signaling and reduced TNFalpha and INFgamma signaling in HTICS+ HER2+:ERα- patients

While we tend to view signature-negative and signature-positive patients as close variants of the same tumor subtype (e.g. HER2^+^:ERα^−^), they may represent different cancer subtypes that conventional immunohistochemistry with limited markers or microarray profiling (e.g. PAM50) fail to distinguish. Indeed, HTICS may classify seeming related tumors into two different subtypes, e.g. HER2^+^:ERα^−^:HTICS^+^ and HER2^+^:ERα^−^:HTICS^−^, which differ by TIC frequency, immune response, etc. The question is, at what point can one classify tumors as distinct subtypes rather than close variants? Alterations in a single gene can determine response to therapy. For examples, the response of HER2^+^ BC to trastuzumab is strongly affected by phosphorylation /activity of SRC [18], expression of the autophagy gene ATG12 [27] or co-treatment with metformin [28]. Therefore, highly related tumors may be separated into 2 groups when response to a single drug is considered. However, if tumors can be classified into 2 groups based on clinical outcome even without therapy, then, for all intent and purposes, these groups may be viewed as distinct subtypes. Interestingly, we showed that HER2^+^:ERα^−^:HTICS^+^ and HER2^+^:ERα^−^:HTICS^−^ patients have a different outcome even when not treated with chemotherapy [12], and may therefore represent distinct subtypes.

**Figure 5:**
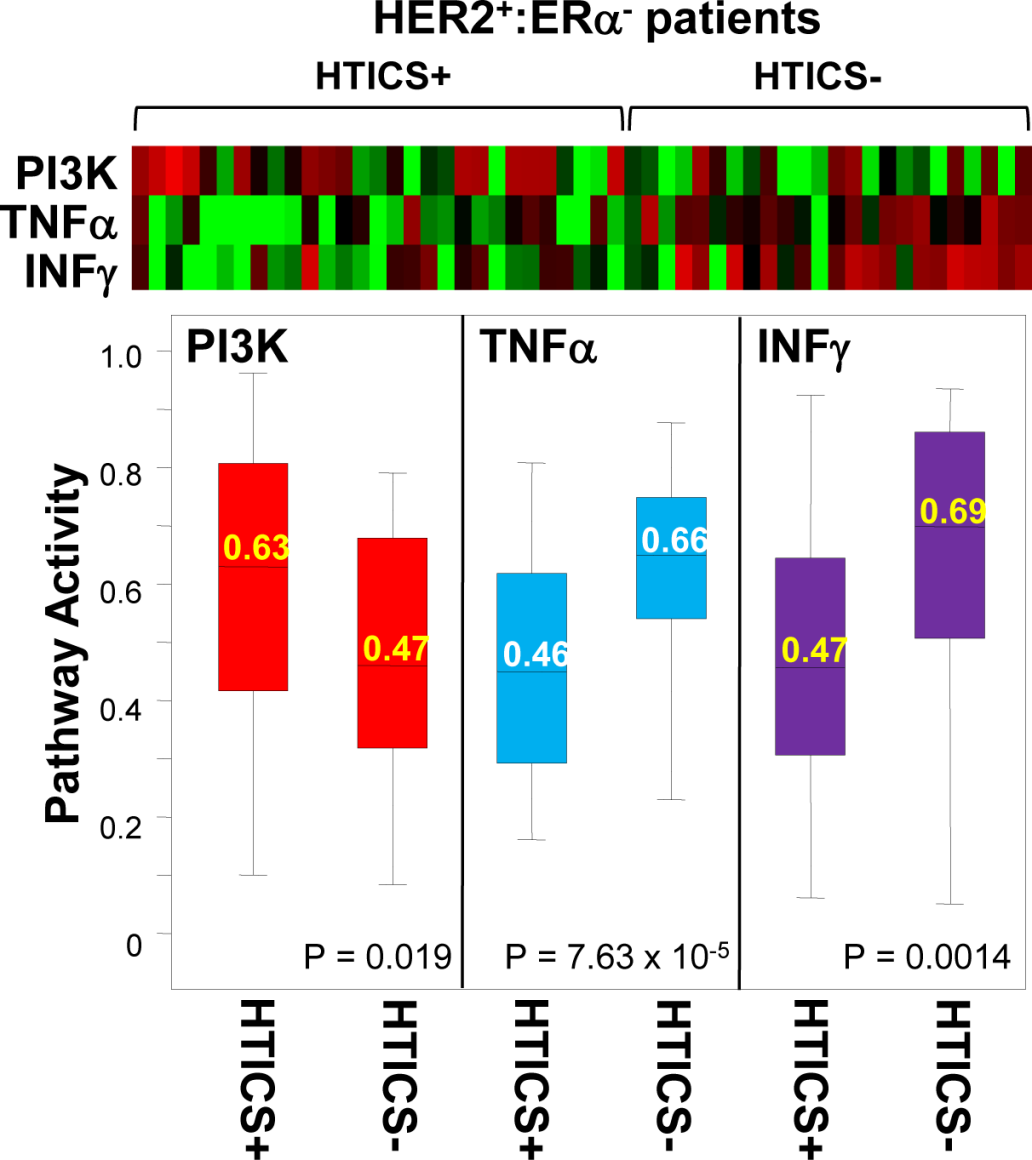
PI3K, TNFalpha and IFNgamma pathway activities are significantly different in HTICS+ versus HTICS- patients Pathway activities calculated for 53 MFS HER2^+^:ER^−^ patients (Figure 2), identified significant differences in activities for PI3K, TNFalpha and IFNgamma pathways in HTICS^+^ versus HTICS^−^ patients. Shown are heat map, box plot distribution, average (labeled in the box) and p values (by t-test).

If signature-negative and signature-positive tumors represent distinct tumor subtypes, what makes them different? With the advent of genome-wide analysis of genetic and epigenetic alterations, this question can now be directly addressed. At a more basic level, one can calculate activity for multiple pathways to identify features that distinguish signature-positive from -negative tumors. We calculated pathway activity for 18 signaling pathways as defined by [29], for 29 HER2^+^:ERα^−^:HTICS^+^ versus 24 HER2^+^:ERα^−^:HTICS^−^ tumors. Strikingly, we found that PI3K pathway activity was significantly elevated in HTICS^+^ versus HTICS^−^ tumors, whereas TNFα and INFγ pathway activities were significantly reduced (Fig. 5). This is in agreement with our HTICS signature analysis where the up-regulated genes are involved in cell proliferation and down-regulated genes are related to immune response (Figure 1B). These results suggest that HTICS^+^ and HTICS^−^ HER2^+^:ERα^−^ tumors are inherently distinguishable, at least in part, by levels of PI3K, TNFalpha and INFgamma signaling. Sequencing genes on these pathways may identify mutations that activate these pathways, separate HER2^+^:ERα^−^ tumors into HTICS^+^ and HTICS^−^ subtypes, and underlie their differential prognosis.

PIK3CA is the second most frequently mutated oncogene whereas the PI3K pathway is frequently activated in BC [30]. Drugs targeting this pathway, including PI3K, AKT, mTOR and autophagy inhibitors, are in different phases of clinical trials and many new drugs are being developed [31]. It was shown that PI3K inhibitors are more effective against tumors with Pik3ca mutations [32]. Our findings therefore suggest that HER2^+^:ERα^−^:HTICS^+^ patients, with elevated PI3K signaling, may be highly responsive to PI3K pathway inhibitors in combination with anti-HER2 and chemotherapy.

### Rationale design of TIC-derived prognostic signatures

When designing TIC-derived prognostic signatures, one should consider the source of tumor samples, identify TICs and address their stability, secure independent cohorts with gene expression and clinical data, optimize a signature on one cohort (training) and then validate on multiple other (test) cohorts. Finally, the signature should be adopted for use on clinical samples. Here we review major steps in this process.

#### 1. Tumor samples

Ideally, a TIC-derived prognostic signature should be derived from related primary tumors. However, this is technically very difficult especially given the heterogeneity of cancer. For example, HER2^+^:ERα^−^ tumors represent ~10% of BC samples. To obtain at least 5-10 independent HER2^+^:ERα^−^ tumors - to identify TICs and derive the signature - one needs fresh biopsies in sufficient quantities from at least 50 patients. Our success in obtaining a good prognostic signature for human cancer using a mouse model for HER2^+^:ERα^−^ BC [33], suggests that this approach may be applicable to other well-defined cancer subtypes. Mouse models of cancer provide ample supply of primary tumors and the ability to identify TICs in an immune-proficient host. Excellent mouse models for very specific breast cancer subtypes are being constantly created (e.g. [34-38]) and they offer great opportunity to generate subtype-specific signatures.

While prognostic signatures are typically derived from primary tumors, it would be interesting to determine whether metastatic lesions could prove a better source. Intuitively, primary tumors should have the upper hand because they harbor the genetic/epigenetic information that determines TIC frequency and metastatic potential. Moreover, the prognostic test will ultimately be performed on primary tumor samples. Yet, given the heterogeneity and parallel evolution of some cancers, primary tumors may not be enriched for TICs that sustain metastatic disease, as has recently been demonstrated in Medulloblastoma [39]. Thus, TICs (or metastatic initiating cells) from metastases may provide better prognostic information than primary tumors.

#### 2. Identification of TICs and their stability

A prerequisite for generation of a TIC-derived signature is the identification of cell surface markers that can distinguish between TICs and non-TICs. In mouse models of mammary adenocarcinomas, CD24 and CD49f, which are used to identify mammary stem cells, are expressed on most tumor cells and cannot be used to segregate TICs from non-TICs. For MMTV-Wnt1 tumors, CD24 and Thy1 were successfully used to identify a small fraction of cells that is highly enriched for TICs [40]. For MMTV-Her2/Neu, surface markers CD49f, Sca-1, CD29, CD90, CD18 and CD14 failed to subdivide the CD24^+^ cell population for enrichment of TICs [41, 42]. We found that JAG1 (and Notch1) could subdivide the CD24 cell population into highly and moderately enriched fractions [12]. The CD24+:JAG1- population contained TICs at a frequency of 1-4.6%. As noted, TIC stability should be determined by comparing multiple secondary tumors derived from single cells to the primary tumors from which they were derived using global gene profiling methods.

#### 3. Cohorts with microarray and clinical data

Success in developing prognostic signatures very much depends on availability of cohorts with clinical and transcript expression data based on RNA extracted from fresh tumor biopsies. For breast cancer, there are relatively large cohorts that are publicly accessible. For many other cancer types, this is a serious limitation. Even for breast cancer, the available cohorts are old and from patients given outdate treatment regimens. This problem is confounded by the fact that one large cohort should be used to “train” the signature, and then at least 2-3 independent cohorts to assess its utility.

#### 4. Bioinformatics

To generate a prognostic signature, a training cohort is used to first determine the clinical relevance of each gene of interest. These genes can be identified through statistical analysis such as ANOVA or SAM by comparing expression in specific cell types (e.g. TIC-enriched vs. non-TICs). Alternatively, all genes on the microarray are used to correlate expression with clinical outcomes. A simple “training” process assesses the association of each individual gene with good or bad clinical outcome using Pearson's correlation or cluster analysis, choosing the most predictive genes [12]. A more sophisticated approach involves binary regression to identify a set of genes with the highest ability to differentiate patients with good/bad prognosis [11, 13]. The advantage of the latter method is that it takes into account the effect of gene combination, rather than individual genes in isolation, to predict clinical outcome. In addition, cross-validation procedures like leave-one-out can be used to test for robustness of a signature. This is done by randomly selecting multiple subsets from the training cohort to gauge signature efficacy [13]. Ultimately, the effectiveness of a signature has to be validated on multiple independent cohorts.

#### 5. Bringing prognostic signatures to the clinic

Because microarray analysis requires large amount of intact RNA, which can only be obtained from freshly biopsied samples, it is not practical to perform this analysis as a diagnostic tool in the clinic. Indeed, most clinical samples are archived as Formalin-Fixed, Paraffin-Embedded (FFPE) tissues. NanoString technology, which does not involve PCR amplification, allows for accurate measurement of gene expression even from degraded RNA samples including FFPE samples at pico molar levels [43]. This technology enables international multi-centered testing with thousands of formalin-fixed specimens, including old archived samples. One limitation of NanoString technology is that unlike microarray analysis in which genes of the entire transcriptome are measured, only a few hundred genes can be monitored at the same time. However, for short signatures such as Oncotype (21 genes) PAM50 (50 genes), NanoString technology has already proved successfully [44], and can be applied to other signatures such as HTICS.

### FUTURE DIRECTIONS

While much effort has been invested in RNA expression-based technologies to generate sensitive prognostic signatures, the advent of exome sequencing at reduced cost (now approaching $1000/sample), opens the door for exome-based prognosis or specific sequencing of genes associated with a particular cancer. This may soon revolutionize diagnosis and treatment at a patient/tumor-specific gene resolution. Sequencing-based prognosis alone may prove to be sufficiently powerful to guide therapy. However, given the complexity of gene regulation, splice form variants, genetic, epigenetic and post-translational effects, we envision that ultimate predictors would be based on complex analysis of several parameters including gene expression and mutation analysis. The applications of such next-generation signatures with effective therapies will likely transform personalized medicine, and dramatically reduce mortality rates in years to come.
